# Adherence to Artesunate-Amodiaquine Therapy for Uncomplicated Malaria in Rural Ghana: A Randomised Trial of Supervised versus Unsupervised Drug Administration

**DOI:** 10.1155/2009/529583

**Published:** 2009-10-21

**Authors:** Kwaku Poku Asante, Ruth Owusu, David Dosoo, Elizabeth Awini, George Adjei, Seeba Amenga Etego, Daniel Chandramohan, Seth Owusu-Agyei

**Affiliations:** ^1^Kintampo Health Research Centre, P.O. Box 200 Kintampo, Brong Ahafo Region, Ghana; ^2^Dodowa Health Research Centre, Ghana Health Service, P.O. Box 1 Dodowa, Dangme West District, Greater Accra Region, Ghana; ^3^Disease Control and Vector Biology Unit/Infectious and Tropical Disease Department, London School of Hygiene & Tropical Medicine, Keppel, WC1E 7HT St London, UK

## Abstract

*Introduction*. To enhance effective treatment, african nations including Ghana changed its malaria treatment policy from monotherapy to combination treatment with artesunate-amodiaquine (AS+AQ). The major challenge to its use in loose form is adherence. *Objective*. The objectives of this study were to investigate adherence and treatment outcome among patients treated with AS+AQ combination therapy for acute uncomplicated malaria. *Methodology*. The study was conducted in two rural districts located in the middle belt of Ghana using quantitative methods. Patients diagnosed with acute uncomplicated malaria as per the Ghana Ministry of Health malaria case definitions were randomly allocated to one of two groups. All patients in both groups were educated about the dose regimen of AS+AQ therapy and the need for adherence. Treatment with AS+AQ was supervised in one group while the other group was not supervised. Adherence was assessed by direct observation of the blister package of AS+AQ left on day 2. *Results*. 401 participants were randomized into the supervised (211) and unsupervised (190) groups. Compliance in both supervised (95.7%) and unsupervised (92.6%) groups were similar (*P* = .18). The commonest side-effects reported on day 2 among both groups were headaches, and body weakness. Parasite clearance by day 28 was >95% in both groups. *Discussion/Conclusions*. Administration of AS-AQ in both groups resulted in high levels of adherence to treatment regimen among adolescent and adult population in central Ghana. It appears that high level of adherence to AS-AQ is achievable through a rigorous education programme during routine clinic visits.

## 1. Introduction

It is estimated that about 2.7 million malaria deaths occur annually with about 90% occurring in Africa [1]. Between 400and 900 million cases ofacute febrile episodes of malaria occur yearly in children living in malaria endemic regions [2]. Those children who do not die may suffer brain damage or experience cognitive and learning deficits [3]. The most important drawback to successful control of malaria is the development of resistance by *Plasmodium* species to commonly-used anti-malarial drugs [4]. Artemisinin-based combination therapy (ACT) has been demonstrated to improve treatment efficacy [5] and reduce the risk of drug resistance and thus recommended by the World Health Organisation as the first line drug for treatment of malaria [6]. Artesunate-amodiaquine (AS-AQ) combination therapy has recently been introduced as the first line drug of choice for the treatment of uncomplicated *P. falciparum* malaria in Ghana [7]. One of the main disadvantages of ACT is achieving good adherence to treatment regimen [8]. In Ghana, prepackaging of antimalarials has been demonstrated to improve adherence and therapeutic outcome [9–11]. However these studies evaluated chloroquine monotherapy for treatment of malaria and not an ACT. It is envisaged that adherence to artesunate-amodiquine (AS+AQ) therapy would be faced with more challenges compared with chloroquine monotherapy because of the perceived side-effects of amodiaquine and limited access to fixed dose combination in the current market.

Adherence to ACTs could be enhanced by treatment supervision by trained community workers. Supervisory home visits by a trained community worker form a major component of the Community Health Planning and Services programme being implemented by the Ghana Health Service [12]. 

This study assessed adherence to AS+AQ therapy and also the effect of adherence to AS+AQ therapy on clinical and parasitological outcomes.

## 2. Methodology

### 2.1. Study Site

The study was carried out in the Kintampo North and South districts of Ghana. The two districts cover in total an area of 7162 km^2^ and are located in the forest-savannah transitional zones in the middle belt of Ghana. The total population based on the Health and Demographic Surveillance System (HDSS) of the Kintampo Health Research Centre (KHRC) was approximately 126000 in the 2007 census (Kintampo Health Research Centre, annual report, 2007) and the main economic base is subsistence farming. The mean monthly temperature ranges from 18°C to 38°C, and the average rainfall is 1250 mm per annum, creating optimal conditions for malaria transmission at a rate of about 269 infective bites per person per year [13].

### 2.2. Study Population

Adolescent and Adult patients (age > 14 years) seen at community-based clinics and diagnosed as having uncomplicated malaria (fever + any level of malaria parasitaemia) were eligible for enrolment into this study. Patients who were unable to tolerate oral therapy, not resident in the study area or unwilling to take part in the study as well as pregnant women, were excluded from the study.

### 2.3. Recruitment

Patients with signs and symptoms were assessed and recorded by a study clinician. Participants who satisfied the eligibility criteria (i.e., any signs and symptoms of malaria + presence of parasitaemia) were recruited into the study and randomised into one of two groups, supervised or unsupervised groups.

### 2.4. Treatment

Artesunate and amodiaquine (IPCA Laboratories, India) were prescribed according to body weight (i.e., bodyweight ≥ 50Kg: 100 mg of artesunate and 300 mg of amodiaquine; bodyweight <50 Kg: 50 mg of artesunate and 150 mg of amodiaquine) and administered twice daily for 72 hours. All participants in each group received the first dose of antimalarial under observation and were educated about AS+AQ dose regimen and the need for adherence as per Ghana's malaria treatment guidelines.

### 2.5. Randomization

Randomization was carried out in blocks of twenty by tossing a coin to determine the first group. The first block of twenty participants was allocated into the supervised group. The subsequent block of twenty participants was allocated into unsupervised group and these were alternated until the desired sample size was achieved. These large blocks were used to prevent overrepresentation of patients coming from the same community allocated to one group.

### 2.6. Follow-up

Participants in the supervised group were visited at their homes by trained community workers to administer subsequent treatment regimen under direct observation on the second and third days of treatment, and post-treatment days (third, seventh, 14, and 28). The participants in the unsupervised group were advised to self-administer the treatment and were visited at home on the third day for assessment of their adherence to the treatment regimen, then on the seventh, 14th, and 28th posttreatment days for parasite clearance and clinical resolution. 

At each visit a symptom assessment questionnaire was used to determine a patient's clinical outcome. Participants found not to be improving were referred to the health facility, reviewed further, and treated by the study clinician.

### 2.7. Laboratory Procedures

Venous blood samples (1 mL) were obtained prior to treatment to determine the haematological, biochemical, and malaria microscopy. Haemoglobin levels were determined using ABX Micros 60 Haematology Analyzer (Horiba ABX, France). Biochemical analyses were determined using Selectra E Clinical Chemistry analyser (Vital Scientific N.V., The Netherlands). Malaria parasite density was estimated from malaria parasite counts per 200 white blood cells and then extrapolated to malaria parasites per microlitre of blood with an assumption that a microlitre of blood contains 8000 leucocytes. Two hundred thick film fields were examined before assigning a negative malaria diagnosis. Prepared blood smears were read by expert microscopy readers in Kintampo Health Research Centre. Ten percent of positive and negative blood smears were randomly selected and confirmed by an independent blinded microscopist.

### 2.8. Outcome Measures

The main outcome measure was adherence to AS+AQ combination therapy. This was defined as the correct number of tablets of artesunate or amodiaquine left on the third day (day 2) of treatment when the treatment regimen was expected to be completed. This was assessed by direct observation of the blister package of artesunate-amodiaquine tablets. Secondary outcome measures included parasite clearance rates on days 14 and 28 determined as the proportion of study participants in each group without *P. falciparum* parasitaemia by microscopy and clinical symptoms reported by day 7 determined as the proportion of study participants in each group with symptoms of malaria reported through a structured questionnaire by day 7.

### 2.9. Sample Size

It was expected that about 65% of study patients would adhere to medication under supervision and about 50% would comply with the treatment without any supervision. A sample size of 182 per arm was estimated given 95% confidence interval and a power of 80. Assuming a 10% drop-out rate, about 202 participants were recruited per group.

### 2.10. Data Analysis

Data were analysed using STATA 9 (StataCorp, College Station TX). Baseline characteristics and the endpoints were compared between the two groups. Point estimates were summarized as means and proportions and interval estimates as ranges and 95% confidence intervals. Biochemical and hematological indices were described according to the CTC AE v.3 grading classification. All statistical tests were two sided, and a *P*-value of ≤ .05 was considered indicative of a statistically significant difference.

### 2.11. Ethical Issues

The study was approved by ethics committees of the Ghana Health Service. The study participants were enrolled after obtaining informed written consent.

## 3. Results

### 3.1. Background Characteristics

**Figure 1:**
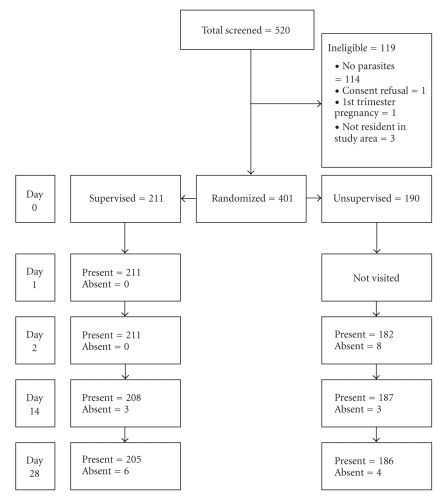
Flow diagram of patient recruitment and follow up.

A total of 520 participants were screened for inclusion into the study. 211 and 190 participants were randomized into the supervised and unsupervised groups, respectively (Figure 1). 

The average age of the participants in the supervised and unsupervised groups were 38.1 and 39.5 years, respectively, and there was no statistically significant difference in the baseline characteristics between the two groups. The mean weight among both groups was 49.6 Kg (48.5–50.7) (Table 1). The mean dosage/day of artesunate was similar 3.1 (95% CI 2.9–3.1) and 3.0 (95% CI 2.9–3.1) mg/Kg body weight for the supervised and unsupervised groups, respectively. The mean dosage for amodiaquine was 9.1 (95% CI 8.8–9.4) and 9.0 (95% CI 8.7–9.3) mg/Kg body weight for the supervised and unsupervised groups, respectively.

### 3.2. Adherence to Artesunate-Amodiaquine Therapy

There was no statistically significant difference in adherence to AS+AQ therapy between the supervised and unsupervised groups (95.7% versus 92.6%; *P* = .18) There was no association between sex, age, or education and adherence to treatment regimen in the supervised and unsupervised groups (Table 2). 

There were no marked differences in the reported side effects within the first one week of treatment between the two groups (Table 3). Headache was the commonest side-effect reported within the first week. The prevalence of headache increased on Day 2 (supervised: 10.6%, unsupervised: 16.4%; *P* = .12) but decreased by Day 7 (supervised: 2.8%, unsupervised: 1.8%; *P* = .20). Body weakness, bodily pains, joint pains, and drowsiness were the other commonly reported side effects after receiving treatment with AS-AQ. There were uncontrollable body movements among 10% and 5.9% of supervised and unsupervised participants, respectively. These were mainly mild uncontrollable extension of the neck and tongue. These cases resolved spontaneously without hospitalization.

### 3.3. Haematological and Biochemical Side-Effects

The proportion of participants with anaemia (hb < 11.0 g/dL) increased significantly between day 0 and day 2 in both groups. The proportion of anaemia among the supervised group increased significantly from 12.6% on day 0 to 38.8% on day 2 (*P* < .01). This pattern was similar in the unsupervised group (15.8% on day 0 to 38.8% on day 2, *P* ≤ .01). Majority of the participants haemoglobin levels decreased by a margin between 0.9 g/dL and 2 g/dL from day 0 to day 2 (supervised = 79.4%, unsupervised = 81.5%  *P* = .64).

Total bilirubin, Aspartate Transaminase (AST), Alanine Transaminase (ALT), Creatinine (Cr), and Urea (Ur) analyses were carried out on day 0 and day 2 to assess liver and renal functions. The proportion of high  (≥ grade 3 on CTC AE V3 classification) biochemical indices were similar among both groups on days 0 and 2 (Table 4).

The proportion of high creatinine levels were similar among both groups on day 0 (supervised = 1.42%, unsupervised  =  0.53%, *P* = .37) and day 2 (supervised  =  11.22%, unsupervised  =  8.74%, *P* = .44). Though the proportion of high (≥ grade 3 on CTC AE V3 classification) creatinine was similar among the two groups at day 0 and 2, there was a significant increase by Day 2 compared with day 0 among both supervised (*P* < .01) and unsupervised (*P* ≤ .01) groups.

### 3.4. Parasite Clearance

There was no difference in the proportion of participants who had parasites cleared on day 28. As per protocol analysis, day 28 parasite clearance was 98% (201/205) among those supervised, compared with 99.5% (185/186) among those unsupervised. Day 28 parasite clearance was 95.3% (201/211) and 97.4% (185/190) among the supervised and unsupervised groups, respectively. PCR was not carried out to distinguish recrudescence from reinfections.

## 4. Discussion

In choosing a new drug or combination of drugs for treatment of malaria, one has to consider several factors such as therapeutic efficacy, side effects, patients' perception about the treatment regimen, cost, and availability and accessibility of the drug(s) in question. While side effects and treatment regimen are key determinants to patient's adherence, perception of illness, treatment-seeking behaviour, and acceptability and affordability of the drug will also influence adherence. Poor treatment practices may lead to suboptimal blood levels or one of the drugs could be given as monotherapy leading to population resistance as occurred with chloroquine. Thus, periodic evaluation of adherence to treatment regimen and optimum ways to ensure adherence among target populations is important.

Adherence to the three-day treatment regimen of AS+AQ was very high in this study in both supervised and unsupervised participants. In a feasibility study of the use of artemether-lumefantrine which has a more complicated three day treatment regimen, >80% of care-givers in southern Ghana correctly administered the drug [14]. A similar study in southern Ghana and other African countries using AS+AQ also demonstrated a high rate of adherence (81%–97%) among caregivers [15]. These result suggests that high levels of adherence to AS+AQ can be achieved in rural communities if an effective education programme on treatment regimen is in place. However, the adherence rates observed in this study may be an overestimation; though the study tried to mimic the routine programme situation in the unsupervised group, the consenting process and home visits by study staff which are not part of the normal practices in the routine malaria case management heath care delivery system could have promoted adherence to therapy beyond what will normally be expected. As part of Ghana's treatment guidelines for uncomplicated malaria with AS+AQ, a prescribing clinician is obliged to explain the treatment regime and possible side-effects to his/her client. This practice was strictly followed in the study and may have influenced the high adherence rates observed. In the training of prescribing clinicians, pharmacist, and chemical sellers, patient education on drug regimen and possible side-effects should be emphasized to ensure adherence. This will particularly be crucial if AS+AQ should in future be deployed for home treatment of malaria as part of the Integrated Management of Childhood Illnesses (IMCI) programme. 

Adherence to treatment is likely to be affected by factors such as age, number of tablets and duration of treatment. In the case of AS+AQ adult patients are expected to take between 12 and 24 tablets of AS+AQ tablets over three days. The introduction of the fixed dose of AS+AQ has halved the number of tablets and this is likely to enhance adherence [16]. However, a study designed to evaluate adherence to the fixed dose of AS+AQ under unsupervised conditions is needed. 

The side-effects reported in this study are similar to those reported in controlled studies [17] with the commonest being headache. Majority of side-effects had resolved within one (1) week of treatment. Body weakness was commonly reported within the first two days posttreatment. Generally, the reported symptoms among this cohort of adults are less than that reported among children who were treated with AS+AQ in northern Ghana [18]. Compliance among participants in both supervised and unsupervised groups was high irrespective of side-effects they experienced during treatment phase. This is consistent with the perception of healing in rural areas ,that is, a period of worsening illness followed by a period of wellbeing. 

Supervision of home treatment of malaria by health workers was perceived to be acceptable among participants in the supervised group. They were willing to allow health workers within their communities to supervise their drug administration. In areas where community-based health programme services (CHPSs) are available, public health nurses could supervise malaria treatment, observe the progress of the sick child, and refer where appropriate. This process will increase parasite clearance and prevent resistance as has been reported among children in Gabon [19]. 

There was a significant increase in the proportion of anaemia among the study participants. A decrease in haemoglobin levels was observed on day 2 following treatment; this was similar to the pattern of haemoglobin dynamics among younger children treated with artemisinin combination drugs (including AS+AQ) in the same area. In these children, there was an initial drop in haemoglobin followed by an increase by day 14 [20]. A similar pattern of haematological recovery post malaria treatment has been reported among other children in northern Ghana [18] and Kenyan children [21]. In this study, haemoglobin levels were determined only on days 0 and 2. Haemoglobin levels are likely to have increased by day 14 as was found in younger children. Further studies are required to understand haematological recovery processes following treatment with artemisinins in adults.

The efficacy of AS+AQ in adult study group in this high malaria transmission area is comparable to AS+AQ efficacy results in other studies where malaria transmission is similarly high: 98.6% obtained in northern Ghana [22], 92.7% in Senegal [23], and 100% in Uganda [24]. 

In conclusion, both supervised and unsupervised administration of AS+AQ resulted in high levels of adherence to treatment regimen among an adult population in a rural district in Ghana. Although this may have been influenced by the intensity of research related activities, it nevertheless gives a good indication of the potential to achieve high level patient adherence through rigorous routine patient education.

*Limitations*. Adherence to therapy was assessed using the number of tablets left in the sachets which is dependent on what participants showed to the investigators. A better method could have been an estimation of drug levels in blood or urine. However, this test is expensive. In the absence of such estimates, the observation method was used. In communities were participants in both groups existed concurrently, adherence levels could have been influenced by contamination. However, this is unlikely due to the short duration between drug treatment and adherence assessment during which sick adolescents and adults are unlikely to move around to cause contamination. Additionally, randomisation was done in blocks of 20 to minimise contamination during the short treatment duration.

##  Contributions

K. P. Asante, R. Owusu, D. Dosoo, S. A. Etego, and S. Owusu-Agyei were involved in the design and implementation of the study. K. P. Asante, R. Owusu, D. Dosoo, E. Awini, G. Adjei, S. A. Etego, and S. Owusu-Agyei contributed to the analysis of the data collected. K. P. Asante, R. Owusu, E. Awini, G. Adjei, D. Chandramohan, and S. Owusu-Agyei contributed to the write up of the paper. All authors read and approved the final manuscript.

## Figures and Tables

**Table 1:** Age, sex, and educational characteristics of study participants.

	Supervised % (*n*)	Unsupervised % (*n*)
Number of respondents	211	190
Mean age, years (95% CI)	38.1 (37.7–40.4),	39.5 (37.1–41.9)
Sex		
Males	34.6 (73)	36.8 (70)
Females	65.4 (138)	63.2 (120)
Educational background		
None	44.5 (94)	53.7 (102)
Primary	20.9 (44)	16.8 (32)
Middle school, JSS	29.4 (62)	27.4 (52)
Technical/commercial/SSS/post Sec	5.2 (11)	2.1 (4)
Physical Examination		
Mean weight, Kg (95% CI)	49.6 (48.5–50.7)	49.7 (48.7–50.7)
Mean temperature (95% CI)	37.9 (37.8–38.0)	38.0 (37.9–38.0)
Biochemical		
Proportion with sexual parasite (%)	2.4 (5/211)	3.2 (6/190)
Mean hemoglobin, g/dL (95% CI)	12.4 (12.1–12.6)	12.4 (11.4–13.3)
Geometric mean ALT, U/L (95% CI)	9.0 (7.9, 10.2)	8.7 (7.6, 10.1)
Geometric mean AST, U/L (95% CI)	35.0 (32.9, 37.1)	35.9 (33.6, 38.3)
Geometric mean TBil, *μ*mol/L (95% CI)	12.2 (11.1, 13.5)	11.6 (10.3, 13.1)
Geometric mean Ure, mmol/L (95% CI)	3.1 (2.9, 3.3)	2.8 (2.6, 3.0)
Geometric mean Cre, *μ*mol/L ( 95% CI)	68.8 (66.2, 71.5)	67.7 (65.4, 70.1)

ALT : Alanine transaminase, AST : Aspartate transaminase, TBil : Total transaminase, Ure : Urea, Cre  =  Creatinine, 95% CI : 95% Confidence Interval.

**Table 2:** Predictors of adherence to artesunate-amodiaqiune treatment.

Factor	Odds ratio (95% CI)	*P*-value*
Group		
Unsupervised	1.00	
Supervised	1.82 (0.76, 4.33)	.18
Sex		
Women	1.00	
Men	0.93 (0.38 2.26)	.87
Age group		
age-groups >20	1.00	
age-groups ≤20	1.25 (0.34, 4.57)	.76
Education		
Education	1.00	
No Educated	1.36 (0.55,3.56)	.51

**Table 3:** Reported side-effects within 7 day post first dose.

Symptom	Day 1*			Day 2			Day 3			Day 7	
	Sup.		Sup.	Unsup.	*P*-value	Sup.	Unsup.	*P*-value	Sup.	Unsup.	*P*-value
			(%)	(%)		(%)	(%)		(%)	(%)	
Headache	11.2	—	10.6	16.4	.12	7.3	11.5	.44	2.8	1.8	.56
Body weakness	15.5	—	13.6	7.9	.20	0.0	0.0	—	0.9	1.1	.87
Bodily pain	8.0	—	9.9	6.5	.22	5.7	8.8	.48	2.5	3.3	.64
Joint pain	6.6	—	8.0	10.4	.43	4.9	10.8	.22	4.0	1.7	.17
Drowsiness	5.1	—	4.2	7.8	.26	0.0	3.3	—	0.0	0.0	—
Uncontrollable body mov't	2.0	—	10.3	5.9	.11	5.4	5.7	.94	2.0	2.2	.89
Nausea	1.0	—	2.9	3.0	.92	4.4	2.7	.57	1.5	3.6	.17
Abdominal pain	2.4	—	3.3	2.1	.46	1.3	1.4	.98	1.9	3.7	.27
Itching	1.4	—	0.0	2.0	—	1.3	0.0	.32	1.9	2.6	.63
Difficulty in sleeping	1.4	—	3.3	1.0	<.01	2.7	1.4	.57	3.3	6.3	.15
Lost of appetite	0.9	—	3.7	3.1	.72	6.6	3.9	.46	3.7	2.6	.51
Diarrhoea	0.0	—	2.3	1.0	.32	0.0	1.4	—	1.8	3.1	.40
Palpitation	0.9	—	1.4	0.5	.38	0.0	0.0	—	0.9	1.0	.89
Rash	0.5	—	0.0	0.0	—	0.0	0.0	—	0.5	0.5	.93
Vomiting	0.0	—	0.5	0.5	.94	0.0	0.0	—	0.0	0.0	—

*Unsupervised group were not assessed on day 1, Sup : supervised group; Unsup : Unsupervised group; Ave : average of supervised and unsupervised groups.

**Table 4:** Proportion of participants with high (≥ grade 3 CTCAE v3 classification) haematological and biochemical indices at Days 0 and 2.

Biochemical assessment		Day 0			Day 2	
	Supervised	Unsupervised	*P*-value	Supervised	Unsupervised	*P*-value
	*n*(%)	*n*(%)		*n*(%)	*n*(%)	
Hemoglobin	1 (0.47)	3 (1.58)	.26	3 (1.42)	5 (2.63)	.07
Alanine transaminase	1 (0.54)	1 (0.60)	.94	1 (0.54)	1 (0.60)	.94
Aspartate transaminase	1 (0.48)	2 (1.06)	.50	0 (0.00)	3 (1.96)	—
Total bilirubin	4 (1.96)	3 (1.62)	.25	4 (2.06)	7 (4.02)	.27
Urea	1 (0.48)	1 (0.53)	.94	7 (3.72)	4 (2.34)	.45
Creatinine	3 (1.42)	1 (0.53)	.37	23 (11.22)	16 (8.74)	.44

Note: The proportion of high creatinine was similar among the two groups at days 0 and 2; there was a significant increase by Day 2 compared with day 0 among both supervised (*P* < .01) and unsupervised (*P* ≤ .01) groups.
